# Mapping Canadian Men’s Recent and Intended Health Behavior Changes Through the Don’t Change Much Electronic Health Program

**DOI:** 10.2196/16174

**Published:** 2020-05-15

**Authors:** John L Oliffe, Nick Black, Jeffrey Yiu, Ryan K Flannigan, Donald R McCreary, S Larry Goldenberg

**Affiliations:** 1 School of Nursing Faculty of Applied Science University of British Columbia Vancouver, BC Canada; 2 Department of Nursing University of Melbourne Melbourne Australia; 3 Intensions Consulting Inc Vancouver, BC Canada; 4 Department of Urologic Sciences University of British Columbia Vancouver, BC Canada; 5 Department of Urology Weill Cornell Medicine New York, NY United States; 6 Department of Psychology Brock University St. Catharines, ON Canada

**Keywords:** men’s e-Health, men’s health behavior change, men’s health promotion

## Abstract

**Background:**

Although evaluation studies confirm the strong potential of men’s electronic health (eHealth) programs, there have been calls to more fully understand acceptability, engagement, and behavior change to guide future work. Relatedly, mapping of behavior changes using health promotion theories including the transtheoretical model (or stages of change) has been recommended to build a translatable empirical base to advance design and evaluation considerations for men’s eHealth programs.

**Objective:**

This study aimed to use a benchmark sample as a reference group to map the recent and intended health behavior changes in Canadian men who use the *Don’t Change Much* (DCM) eHealth program. The hypothesis being tested was that increased exposure to DCM would be positively associated with men’s recent and intended health behavior changes.

**Methods:**

DCM users (n=863) were sampled for demographic data and self-reported recent and intended health behavior changes. Respondents also reported their usage (frequency and duration) for each of the 3 DCM components (web, newsletter, and social media) and were allocated to limited exposure (257/863, 29.8%), low exposure (431/863, 49.9%), and high exposure (175/863, 20.3%) subgroups. A benchmark sample (n=2000), comprising respondents who had not accessed DCM provided a reference group. Bivariate analysis of recent and intended health behavior changes and DCM exposure levels were used to compute the strength of association between the independent variables (exposure levels) and the 10 categorical dependent variables (recent and intended health behavior changes). Binary logistic regression models were computed for each of the 10 recent and intended health behavior changes. Linear regression was used to model the association between the number of recent and intended changes and the level of exposure to DCM.

**Results:**

Compared with the benchmark reference group, DCM high-exposure respondents had significantly increased odds for 9 of the 10 health behavior changes, with the largest effect size observed for Changed diet or Improved eating habits (odds ratio [OR] 5.628, 95% CI 3.932-8.055). High-exposure respondents also had significantly increased odds for 9 intended health changes, with the largest effect sizes observed for Reduce stress level (OR 4.282, 95% CI 3.086-5.941). Moderate effect size (goodness of fit) was observed for increased total number of recent (F_12,2850_=25.52; *P*.001; adjusted *R*^2^=.093) and intended health behavior changes (F_12,2850_=36.30; *P*.001; adjusted *R*^2^=.129) among high-exposure respondents.

**Conclusions:**

DCM respondents contrasted the predominately precontemplative benchmark sample mapping across the contemplative, preparation, and action stages of the transtheoretical health behavior change model. Almost 10% of variation in the recent and 13% of variation in the intended health behavior changes can be explained by DCM exposure and demographic factors, indicating the acceptability of this men’s eHealth resource.

## Introduction

The case for men’s health is often articulated through sex differences research wherein lower life expectancy in men (compared with that of women) is connected to their overall poor self-health practices, including estrangement from professional *in person* health care services [1,2]. The major mortality causes accounting for men’s reduced life expectancy include cardiovascular disease, suicide, motor vehicle accidents, liver failure (most often due to alcohol overuse), and infectious diseases (most often HIV) [3]. These (and many other) mortality causes and contributors to morbidities are seemingly amenable to prevention-based interventions, and by virtue of that, tailored health promotion programs have surfaced to garner men’s health behavior changes [4-6]. The platforms and mechanisms for promoting men’s health, although diverse, have grown exponentially in the electronic health (eHealth) sector over the last two decades [7,8]. That said, empirical insights to the acceptability, engagement levels, and behavior changes reaped through these well-intended men’s eHealth programs, while promising, are emergent and drawn from diverse study designs [7,9]. The aim of this study was to use a benchmark sample as a reference group to map the recent and intended health behavior changes in Canadian men who use the *Don’t Change Much* (DCM) eHealth program [10]. The hypothesis being tested was that increased DCM exposure levels would be positively associated with men’s recent and intended health behavior changes.

Men’s lifestyles have attracted health promotion research describing specific risk factors and a range of potential remedies. In terms of risk, unhealthy diets, alcohol overuse, smoking, and sedentary lifestyles have featured as issues warranting tailored behavior change interventions [11]. There is also diversity in the predisposition to the aforementioned behavior risks whereby inequities within social determinants of health (ie, income, employment, education) result in disadvantage to some male subgroups both in terms of their knowledge levels and access to health care services [12,13]. Within this context, men’s health risks and the potential for behavior change emerge as somewhat relative, deeply reliant on resources being freely available and easily accessible. By lessening structural barriers and mobilizing men’s strength-based efforts for optimizing their health, important behavior changes can occur for men [13]. In this regard, men’s eHealth resources have great potential to improve access and meet many health promotion needs for men.

Men’s eHealth programs have grown significantly to deliver diverse information and services across ever expansive platforms (ie, web, social media, and email). The wide variety of men’s eHealth programs include interventions tailored to address weight loss [14], smoking cessation [15], prenatal health education [16], fathering [17], depression management [7,18], sexual health [19-24], and prostate cancer [25]. Although there are claims that eHealth resources can engage diverse subgroups of men, satiating their preferences for anonymity and self-directed health help-seeking, two significant issues prevail. The first relates to the varied conceptualizations and approaches to evaluating needs analyses (acceptability) and end-user engagement, as well as the lack of conclusive empirical evidence regarding associations between men’s eHealth programs and behavior change. For example, needs analysis of men transitioning to fatherhood by Da Costa et al [17] confirmed substantial interest among new and expectant fathers for using internet-delivered strategies to promote their mental health and prepare for parenthood. Within this and similar needs analyses work, the insights drawn from potential end users have affirmed the acceptability of men’s eHealth programs in specific contexts and informed tailored content and targeted delivery of the interventions. Evaluations of newly launched and established eHealth programs have tended to focus on men’s engagement or linkages to behavior change [16,22]. Examples include a single-group, repeated measures design evaluating the applicability of Man Central (a web and mobile phone intervention for men with depression), which showed significant improvements in depression symptoms, depression risk, externalizing symptoms, and work and social functioning among end users [7]. Pre-post eHealth evaluation studies reported significant improvements in depressive symptoms among a community sample of overweight and obese men with depression [14], whereas Bottorff et al [15] highlighted tobacco reduction and/or cessation for 66% (43) of men who used the QuitNowMen resource. Klein et al’s [24] quasi-experimental, 2-arm study evaluated *Real Talk* (an eHealth harm reduction intervention targeting black men who have sex with men) reported end user’s HIV knowledge gains (although there were no significant differences between *Real Talk* and the control participant’s actual condom use or other risk reduction strategies). Although the aforementioned and many other men’s eHealth evaluation studies have been limited by small samples, attrition, and/or a lack of control groups, the results are encouraging [25,26]. In sum, acceptability and evaluation studies confirm the strong potential of men’s eHealth programs amid calls for more evidence to efficiently guide future work and confidently claim sustained health behavior change effects.

The second issue relates to incorporating theory to map men’s health behavior changes and their relationships to tailored eHealth programs. Simoni et al [26] argued the need for health promotion theory in men’s eHealth to build a translatable empirical base and advance the design and evaluation of gender-sensitized interventions. Among many health promotion theories, the transtheoretical (or stages of change) model [27,28] has guided eHealth program designs and helped contextualize barriers and facilitators to men’s health behavior changes in a range of contexts [29-31]. Comprising precontemplative, contemplative, preparation, action, and maintenance, these interconnected and recursive stages of change characterize the transtheoretical model, underpinned by processes reliant on men’s access, self-efficacy and recognition of, and commitment to the benefits of sustained modifications. Program design, acceptability, engagement, and behavior change evaluations of men’s eHealth resources can, and many argue should, interface with the transtheoretical model (or similar) to map men’s progress and adjust intervention content and/or delivery accordingly [29-31]. In line with this recommendation, and reflecting our commitment to fully integrating the transtheoretical model, this study adds to methodological approaches by using a benchmark sample as a reference group to map men’s eHealth engagement and behavior change, in making recommendations for DCM, and, more broadly, the burgeoning field of men’s eHealth.

## Methods

### Overview

Following university ethics approvals, demographic and self-reported recent and intended health behavior change data were collected via survey questionnaires from two Canadian male cohorts: (1) benchmark (reference group), comprising respondents who had not accessed DCM, and (2) DCM users. Data collection details and cross-sectional findings from the benchmark sample have been reported elsewhere [6,32]. Briefly, the 5083 respondents recruited via web-based panel provider to complete a Canadian men’s health survey were reduced to 2000, stratified by age and location to be close to the most recent Canadian census data. The 15-min web benchmark survey was administered from April 20, 2017, to April 28, 2017.

The second cohort comprised DCM users recruited via the DCM website and email newsletter recipient list, and these respondents were incentivized with the option to enter a prize draw to win Can $500 (US $377.60) cash. By way of background, the DCM eHealth program was purpose-built in 2014 by the Canadian Men’s Health Foundation, a national nonprofit organization, with the goal of inspiring men and their families to lead healthier lives. Reliable information and easily accessible tips are developed based on focus group interviews with Canadian men and reviews of the men’s health literature, and by drawing from the expertise of global thought leaders. The DCM information shared through testimonials, text, video, and audio is brief, often times humorous, and always easy to access with a focus on practical strategies to improve diet, exercise, sleep, and stress management as well as reduce alcohol use and/or smoking. A range of strategies are offered in point form recognizing that end users are diverse in their contexts, needs, and alignments to the transtheoretical stages of change (ie, precontemplative, contemplative, preparation, action, and maintenance). In addition to providing a framework for evaluating DCM, the program content was developed drawing on the transtheoretical model. Specifically, DCM’s strength-based approach and wide-reaching materials were purpose-built to engage men at diverse levels of readiness to change and progress points. In essence, the interconnected stages characterizing the transtheoretical model guided the DCM content design to work with men at whatever point they were at, to advance their self-health.

The DCM demographic and survey questionnaire data were collected between January 1, 2018, and March 31, 2018; the data collection tools were identical to those of the benchmark survey but also included questions about the respondent’s usage (duration and frequency) of the three DCM components (web, newsletter, and social media). Of the 1743 respondents who went to the DCM survey introduction page, 94.8% (1653) opted in. This sample was reduced to 1034 Canadian male DCM users by removing incomplete surveys (n=459), female respondents (n=90), speeding and/or straight lining responses (n=60), respondents from outside of Canada (n=7), and those under the age of 19 years (n=3). Some demographics were asked at the end of the survey (education, household income, and ethnicity), making it impossible to accurately evaluate and report the demographic characteristics for the 459 incomplete surveys. Speeding was assigned to respondents who completed the survey in 5 min or less (less than one-third of the median completion time), and straight lining comprised respondents inputting the same numerical response to all Likert items on 2 or more consecutive survey pages. The final sample of 863 DCM users was obtained through listwise removal of an additional 171 respondents who answered *not sure* to questions regarding their use (duration and frequency) of the 3 DCM components, as a numerical code could not be assigned to classify those respondents’ DCM exposure level.

### Measures

Demographic data, including age, employment, living arrangements (lives alone; children younger than 19 years living at home), education, visible minority, sexual orientation, and household income (before taxes) were collected. *Visible minority* is the terminology used by Statistics Canada, and based on that classification system, we used the term in our postcoding. In addition to being a Canadian resident, respondents were asked: *Do you belong to any distinct ethnic or cultural group?* with response options: *Yes (please specify)* or *No (Prefer not to say)*. Responses from those who answered *Yes (please specify)* were postcoded using Statistics Canada 2016 Census definitions. Regards sexual orientation, respondents were asked the following: *Do you consider yourself to be: Select one: Heterosexual or straight, Gay or lesbian, Bisexual, Not sure or questioning, or Other (please specify)*. Responses were coded as a dichotomous variable (0=heterosexual or straight, 1=other).

Respondents in both cohorts were also asked about the recent and intended changes to improve their health. The first question asked, *In the past 12-months, have you made any changes that would improve your health?* inviting respondents to select all that applied from the following 10 items: (1) changed diet or improved eating habits, (2) made an effort to sit less and walk more, (3) increased exercise, sports or physical activity, (4) I haven’t made any changes, (5) drink less alcohol, (6) had a routine check-up or visit to doctor, (7) improved consistent sleep quality, (8) lost weight, (9) reduced stress level, and (10) quit or reduced smoking. Intended changes were collected through soliciting responses to the same 10 options, with the stem question, *In the next month (30-days), do you intend to make any changes that would improve your health? Select all that apply*.

The 863 DCM respondents had access to three DCM eHealth components: (1) the website, (2) email newsletter, and (3) social media accounts (Facebook, Instagram, and Twitter). Respondents checked yes, no or not sure options for each of the three DCM components in answering the question, *Have you ever used, or subscribed to, any of the following DCM resources?* Respondents who checked *Not sure* responses were excluded. *No* responses were allocated a zero value and assigned to the limited exposure subgroup. *Yes* responses were used to subsequently populate DCM items (website, newsletter, and social media) in asking 2 additional questions to score each respondent’s DCM usage (duration and frequency) and calculating exposure levels [33]. The work of Quinn and Chaudoir [33] guided the assignment of numerical codes to participant’s single-item frequency and duration responses for the DCM components that they had used.

1. *When did you FIRST use, or subscribe to, the following resources?* Response options: never before, in the past month, 1-6 months ago, 7-12 months ago, 13-24 months ago, more than 2 years ago, and not sure. Participants checked the duration level based on their first use for the DCM resources. The categorical measure was converted to a continuous measure representing the duration as follows: *never before*=0.0, *in the past month*=0.5, *1-6 months ago*=3.5, *7-12 months ago*=9.5, *13-24 months ago*=18.5, *more than 2 years ago*=24.0, and not sure (excluded).
2. *How often do you use or access the following resources?* Response options were as follows: several times a day, once a day, several times a week, once a week, several times a month, once a month, several times a year, once a year, less often, do not use, and not sure. To calculate frequency, the checked categorical measure was converted into a continuous measure: *several times a day*=1095, *once a day*=365, *several times a week*=156, once a week=52, several times a month=36, *once a month*=12, *several times a year*=3, *once a year*=1, *less often* =0.5, *do not use*=0, and not sure (excluded).

DCM user classifications were based on the sum of the product of duration and frequency for each of the three DCM components, to calculate respondent exposure scores. Duration responses were converted to numerical scores that represented the number of months since accessing DCM. Frequency responses were converted to numerical scores proportional to the number of times each DCM component was used. To illustrate, respondents who completed the survey questionnaire but indicated a duration of *Never before* and a frequency of *Do not use* for all three DCM components received a summed score of 0 and were classified to the limited-exposure group (257/863, 29.8%). A user who engaged one DCM resource for a duration of *In the past month* (0.5) at a frequency of *Less often* (0.5) received a total summed score of 0.25 and was classified to the low-exposure subgroup (431/863, 49.9%; range 0.25-680). A respondent who used two DCM programs, both for a duration of *7-12 months ago* (9.5) at a frequency of *Several times a month* (36), received a total summed score of 684 and was classified as high exposure (175/863, 20.3%; range 684-9048). Through these classifications, three DCM user subgroups, limited, low, and high exposure, were delineated.

### Data Analysis

Bivariate analyses were conducted to assess the magnitude of differences in demographic factors between benchmark respondents and DCM users. The chi-square test was used to assess whether there was an association between the two categorical variables. Cohen *d* was used to calculate effect size. When the means of three or more independent groups were compared, Cohen *d* was obtained by computing partial eta-squared (another type of effect size) and then converting partial eta-squared to Cohen *d* using formulae in the study by Cohen [34]. Cramer’s V was used to calculate an effect size for the strength of association between the two categorical variables, with values ranging from 0 to 1 (inclusive) [35].

Regression analysis included logistic regression to model the association between the dependent variables recent and intended health behavior changes, and independent variable, level of exposure to DCM. R-squared values were used to measure the proportion of variance in a dependent variable that can be explained by the independent variable in the regression models [34]. For models analyzing recent changes, the dependent variable was whether the user made the specified behavior change (eg, *Increased exercise, sports or physical activity*) in the past 12 months or not. Similarly, for models analyzing intended changes, the dependent variable was whether the user intended to make the specified behavior change (eg*, Change diet or improve eating habits*) in the next month (30 days) or not. Linear regression was used to model the association between the number of recent and intended changes and the level of exposure to DCM. In total, two linear regression models were computed, with the first model having the number of recent changes as the dependent variable, and the second model having the number of intended changes as the dependent variable. The DCM users were classified into 1 of 3 categories, limited, low, or high exposure. Benchmark respondents, classified as no exposure, were the reference group. All logistic and linear regression models controlled for the following covariates: age, employment, living arrangements (lives with partner; children younger than 19 years living at home), education, visible minority, sexual orientation, and household income (before taxes). Odds ratios are effect sizes and were used to indicate the strength of association between predictor variables and dichotomous outcome variables [36]. Variance inflation factors (VIFs) were computed as a collinearity diagnostic check.

## Results

Benchmark respondents had not used DCM and served as a reference group to assess the effect of DCM program exposures on recent and intended health behavior changes. Assessment of the magnitude of differences in demographic factors between benchmark respondents and DCM users revealed small to negligible effect sizes for the 8 demographic factors (see Table 1). Most respondents in both cohorts reported being employed, living with a partner, not living with children younger than 19 years, not having graduated from university, and identifying as heterosexual. There were no statistically significant differences in age between the two cohorts. Compared with benchmark respondents, a higher proportion of DCM users reported having a household income of Can $120,000 (US $92,307.69) or more, although household income and the two cohorts were weakly associated as a whole.

For the DCM group, all recent health behavior changes had significant associations with increased levels of exposure to DCM. Moderate effect sizes were observed in *Changed diet or improved eating habits* (χ^2^_3_=210; *P<*.001; Cramer’s V=0.271) and *Made an effort to sit less and walk more* (χ^2^_3_=167; *P*<.001; Cramer’s V=0.242). All intended health behavior changes had significant associations with increased DCM exposure. Moderate effect sizes were observed for the following seven intended health behavior changes: (1) *Improve consistent sleep quality* (χ^2^_3_=207; *P<*.001; Cramer’s V=0.269), (2) *Changed diet or improved eating habits* (χ^2^_3_=181; *P<*.001; Cramer’s V=0.252), (3) *Increase exercise, sports or physical activity* (χ^2^_3_=169; *P<*.001; Cramer’s V=0.243), (4) *Make an effort to sit less and walk more* (χ^2^_3_=168; *P*<.001; Cramer’s V=0.242), (5) *I don’t intend to make any changes* (χ^2^_3_=148; *P<*.001; Cramer’s V=0.227), (6) *Reduce stress levels* (χ^2^_3_=129; *P<*.001; Cramer’s V=0.212), and (7) *Lose weight* (χ^2^_3_=119; *P<*.001; Cramer’s V=0.205). There was a significant association between the total number of recent health changes and increased DCM exposure levels (ranging no exposure 2.39 to 4.23 high exposure), with a large effect size observed (*P*<.001; *d*=0.818). There was a significant association between the total number of intended health changes and increased DCM exposure levels (ranging no exposure 2.09 to 3.92 high exposure), with a very large effect size observed (*P*<.001; *d*=1.018). Following Bonferroni-adjusted Dunn pairwise tests, with the no-exposure benchmark cohort, all recent and intended changes in the limited-, low-, and high-exposure DCM subgroups were statistically significant (*P*<.001). Recent changes were also statistically significant for DCM limited- and high-exposure subgroups (*P*<.001), as well as for the DCM low- and high-exposure subgroups (*P*=.01).

**Table 1:** Benchmark versus Don’t Change Much users’ sample profile.

Demographics and baseline characteristics			Benchmark (n=2000)		DCM^a^ users (n=863)		Chi square (*df*)		*P* value		Cramer’s V
Age, mean (SD)			46.99 (15.67)		47.34 (11.96)		−0.646^b^		.52		.024^c^
**Employed, n (%)**			N/A^d^		N/A		51.319 (1)		<.001		0.134
	Yes	1307 (65.4)		680 (78.8)		N/A		N/A		N/A	
	No	693 (34.6)		183 (21.2)		N/A		N/A		N/A	
**Partner living with respondent, n (%)**			N/A		N/A		17.947 (1)		<.001		0.079
	Yes	1210 (60.5)		594 (68.8)		N/A		N/A		N/A	
	No	790 (39.5)		269 (31.2)		N/A		N/A		N/A	
**Children <19 years living with respondent, n (%)**			N/A		N/A		80.292 (1)		<.001		0.167
	Yes	441 (22.0)		330 (38.2)		N/A		N/A		N/A	
	No	1559 (78.0)		533 (61.8)		N/A		N/A		N/A	
**Highest level of education, n (%)**			N/A		N/A		9.234 (1)		.002		0.057
	Graduated university	823 (41.2)		408 (47.3)		N/A		N/A		N/A	
	Other	1177 (58.8)		455 (52.7)		N/A		N/A		N/A	
**Visible minority, n (%)**							.128 (1)		.72		0.007
	Yes	218 (10.9)		98 (11.4)		N/A		N/A		N/A	
	No	1782 (89.1)		765 (88.6)		N/A		N/A		N/A	
**Sexual orientation, n (%)**			N/A		N/A		6.562 (1)		.01		0.048
	Heterosexual	1805 (90.2)		751 (87.0)		N/A		N/A		N/A	
	Gay, bisexual, questioning, other	195 (9.8)		112 (13.0)		N/A		N/A		N/A	
**Household income, n (%)**			N/A		N/A		31.505 (2)		<.001		0.105
	Can $59,999 or less (US $46,153.07 or less)	747 (37.4)		238 (27.6)		N/A		N/A		N/A	
	Can $60,000 to $119,999 (US $46,153.86–92,306.92)	855 (42.8)		392 (45.4)		N/A		N/A		N/A	
	Can $120,000 or more (US $92,307.69 or more)	398 (19.9)		233 (27.0)		N/A		N/A		N/A	

^a^DCM: Don’t Change Much.

^b^As a ratio variable, the test performed was a *t* test for this characteristic.

^c^As a ratio variable, the test performed was a Cohen *d* for this characteristic.

^d^N/A: not applicable.

Compared with the benchmark no-exposure respondents, high-exposure respondents had significantly increased odds for all recent health behavior changes except *Quit or reduced smoking* (OR 0.820, 95% CI 0.461-1.458) and significantly decreased odds for *I haven’t made any changes* (OR 0.140, 95% CI 0.065-0.301) while holding other predictor variables constant. Moderate effect sizes were observed for *Changed diet or improved eating habits* (OR 5.628, 95% CI 3.932-8.055), *Increased exercise, sports or physical activity* (OR 3.439, 95% CI 2.444-4.839), and *I haven’t made any changes* (OR 0.140, 95% CI 0.065-0.301) [36]. Of the controlled predictor variables, age, employment, lives with children, education, and income were statistically significant with small effect sizes for some of the recent health behavior changes (see Table 2; full table in Multimedia Appendix 1).

**Table 2:** Logistic regressions between statistically significant demographics and recent health changes (separate multiple logistic regressions were conducted for each outcome variable with all predictor variables entered on the same step).

Dependent variables (recent health change)	Predictor variables, odds ratio (95% CI)											
	Don’t Change Much level of exposure (ref=benchmark no exposure)				Age (years)		Employment		Education		Household income (ref=Can $60,000-$119,999; US $46,153.86–$92,306.92)	
	Limited exposure	Low exposure	High exposure							$59,999 or less (US $46,153.07 or less)		$120,000 or more (US $92,307.69 or more)
Changed diet or improved eating habits	2.008 (1.541-2.616)^a^	3.228 (2.588-4.027)^a^	5.628 (3.932-8.055)^a^	1.009 (1.002-1.015)^b^		1.136 (0.933-1.382)		0.792 (0.672-0.933)^b^		0.887 (0.734-1.074)		0.955 (0.776-1.175)
Made an effort to sit less and walk more	1.892 (1.442-2.481)^a^	3.079 (2.472-3.835)^a^	3.39 (2.457-4.678)^a^	1.018 (1.012-1.025)^a^		1.109 (0.902-1.363)		1.058 (0.893-1.253)		1.063 (0.87-1.299)		1.358 (1.101-1.676)^b^
Increased exercise, sports or physical activity	1.822 (1.398-2.375)^c^	2.064 (1.661-2.564)^c^	3.439 (2.444-4.839)^c^	0.992 (0.986-0.999)^a^		0.898 (0.743-1.086)		1.079 (0.921-1.265)		0.846 (0.703-1.018)		1.273 (1.042-1.557)^a^
I have not made any changes	0.39 (0.256-0.595)^c^	0.23 (0.154-0.344)^c^	0.14 (0.065-0.301)^c^	0.994 (0.986-1.002)		1.142 (0.893-1.461)		1.153 (0.936-1.421)		1.09 (0.857-1.385)		1.091 (0.834-1.426)
Drank less alcohol	1.486 (1.108-1.994)^b^	1.822 (1.439-2.306)^c^	3.287 (2.375-4.549)^c^	0.993 (0.986-1)		0.95 (0.765-1.179)		0.803 (0.668-0.964)^a^		1.031 (0.836-1.272)		0.876 (0.692-1.109)
Had a routine check-up or visit to doctor	1.855 (1.399-2.46)^c^	2.129 (1.693-2.677)^c^	2.082 (1.497-2.896)^c^	1.049 (1.041-1.056)^c^		0.572 (0.466-0.703)^c^		0.924 (0.777-1.1)		0.86 (0.7-1.056)		1.138 (0.919-1.41)
Improved consistent sleep quality	1.383 (1.014-1.885)^a^	1.781 (1.397-2.27)^c^	2.376 (1.696-3.329)^c^	1.001 (0.993-1.008)		1.014 (0.807-1.275)		0.994 (0.822-1.202)		1.113 (0.892-1.39)		1.051 (0.826-1.337)
Lost weight	1.255 (0.949-1.66)	1.602 (1.284-2.001)^c^	2.186 (1.59-3.005)^c^	1.002 (0.995-1.008)		1.106 (0.902-1.356)		0.84 (0.709-0.995)^a^		0.928 (0.761-1.131)		1.095 (0.887-1.352)
Reduced stress level	1.035 (0.758-1.415)	1.651 (1.305-2.089)^c^	1.945 (1.393-2.716)^c^	1.003 (0.996-1.01)		0.884 (0.712-1.097)		0.981 (0.818-1.177)		1.244 (1.007-1.537)^a^		0.981 (0.777-1.24)
Quit or reduced amount smoked	1.652 (1.162-2.347)^b^	0.944 (0.66-1.35)	0.82 (0.461-1.458)	0.988 (0.979-0.997)^b^		1.369 (1.025-1.827) ^a^		0.447 (0.341-0.585)^c^		1.316 (1.006-1.721)^a^		0.6 (0.413-0.873)^b^

^a^*P*<.05.

^b^*P*<.01.

^c^*P*<.001.

Compared with the no-exposure respondents, high-exposure respondents had significantly increased odds for all intended health changes except *Quit or reduce amount smoked* (OR 1.043, 95% CI 0.559-1.946), while holding other predictor variables constant. Moderate effect sizes were observed for *Reduce stress level* (OR 4.282, 95% CI 3.086-5.941), *Improve consistent sleep quality* (OR 4.019, 95% CI 2.911-4.547), *Increase exercise, sports or physical activity* (OR 3.649, 95% CI 2.565-5.191), and *I don’t intend to make any changes* (OR 0.162, 95% CI 0.082-0.321; see Table 3; full table in Multimedia Appendix 2).

A linear regression was, then, conducted. Compared with the no-exposure respondents, high-exposure respondents had significantly increased total number of recent and intended health changes, while holding other predictor variables constant. Moderate effect size (goodness of fit) was observed for both total number of recent health behavior changes (*F*_12,2850_=25.52; *P*<.001; adjusted *R*^2^=.093) and intended health behavior changes (*F*_12,2850_=36.30; *P*<.001; adjusted *R*^2^ =.129). The VIF criterion was within the acceptable range, and there was no indication of multicollinearity.

**Table 3:** Logistic regressions between statistically significant demographics and intended health changes (separate multiple logistic regressions were conducted for each outcome variable with all predictor variables entered on the same step).

Dependent variables (intended health change)	Predictor variables, odds ratio (95% CI)								
	Don’t Change Much usage (ref=benchmark no exposure)			Age (years)	Employment	Education	Visible minority	Household income (ref=Can $60,000-$119,999; US $46,153.86–$92,306.92)	
	Limited exposure	Low exposure	High exposure					$59,999 or less (US $46,153.07 or less)	$120,000 or more (US $92,307.69 or more)
Improve consistent sleep quality	3.018 (2.3-3.959)^a^	3.633 (2.905-4.543) ^a^	4.019 (2.911-5.547) ^a^	0.992 (0.985-0.999)^b^	1.003 (0.81-1.242)	0.84 (0.703-1.004)	1.311 (1.008-1.706) ^b^	0.961 (0.781-1.182)	1.019 (0.814-1.275)
Change diet or improve eating habits	3.671 (2.805-4.805)^a^	2.612 (2.097-3.254)^a^	3.244 (2.356-4.465) ^a^	0.992 (0.985-0.998)^b^	0.94 (0.765-1.154)	0.826 (0.696-0.98)^b^	1.261 (0.976-1.629)	0.866 (0.709-1.058)	0.945 (0.762-1.172)
Increase exercise, sports or physical activity	2.707 (2.048-3.579) ^a^	2.934 (2.336-3.687) ^a^	3.649 (2.565-5.191) ^a^	0.994 (0.988-1)^b^	1.029 (0.85-1.245)	0.928 (0.79-1.09)	1.295 (1.01-1.661)^b^	1.037 (0.86-1.249)	1.109 (0.904-1.361)
Make an effort to sit less and walk more	2.837 (2.168-3.713) ^a^	3.253 (2.607-4.061) ^a^	2.954 (2.144-4.069) ^a^	1.013 (1.006-1.019) ^a^	0.748 (0.61-0.918)^c^	0.816 (0.687-0.969)^b^	0.858 (0.652-1.128)	0.973 (0.796-1.189)	1.105 (0.891-1.371)
I don’t intend to make any changes	0.223 (0.135-0.369)^a^	0.143 (0.089-0.23)^a^	0.162 (0.082-0.321)^a^	1.005 (0.997-1.013)	0.943 (0.743-1.195)	1.132 (0.921-1.392)	0.889 (0.642-1.231)	1.063 (0.839-1.347)	1.177 (0.904-1.534)
Reduce stress level	2.401 (1.803-3.197)^a^	2.337 (1.844-2.96)^a^	4.282 (3.086-5.941)^a^	0.983 (0.976-0.99)^a^	1.176 (0.934-1.482)	0.906 (0.751-1.093)	1.046 (0.79-1.384)	0.987 (0.792-1.229)	0.936 (0.738-1.189)
Lose weight	2.641 (2.016-3.458)^a^	2.4 (1.932-2.983)^a^	1.976 (1.44-2.713)^a^	1.005 (0.999-1.011)	1.057 (0.872-1.282)	0.8 (0.68-0.939)^c^	0.867 (0.674-1.114)	0.904 (0.75-1.091)	1.085 (0.886-1.328)
Have a routine check-up or visit to doctor	2.19 (1.624-2.953) ^a^	2.024 (1.57-2.61) ^a^	2.067 (1.443-2.961) ^a^	1.021 (1.013-1.029) ^a^	0.899 (0.71-1.139)	0.895 (0.734-1.092)	1.238 (0.913-1.678)	1.059 (0.842-1.332)	0.812 (0.627-1.05)
Drink less alcohol	1.677 (1.204-2.336)^c^	1.901 (1.453-2.489) ^a^	2.974 (2.077-4.26) ^a^	0.995 (0.987-1.003)	1.01 (0.782-1.304)	0.61 (0.491-0.757) ^a^	1.228 (0.894-1.687)	0.731 (0.569-0.941)^b^	1.058 (0.813-1.376)
Quit or reduce smoking	1.415 (0.932-2.148)	0.829 (0.535-1.286)	1.043 (0.559-1.946)	0.987 (0.977-0.997)^b^	1.241 (0.896-1.72)	0.446 (0.324-0.614) ^a^	0.664 (0.403-1.095)	1.236 (0.909-1.68)	0.504 (0.312-0.816)^b^

^a^*P*<.001.

^b^*P*<.05.

^c^*P*<.01.

## Discussion

### Principal Findings and Comparison With Previous Work

This study’s findings confirm the potential of men’s eHealth programs as previously described in a range of contexts and diverse studies [7,14-25]. Adding to the literature focused on understanding men’s eHealth acceptability, engagement, and behavior change, this study contributes some important empirical insights and supports calls for future research to more fully investigate dose-response relationships with randomized controlled trials [25,26]. Although careful not to overstate the current findings or imply attribution, some explanations and potential implications for the statistically significant associations between men’s DCM exposure levels and their recent and intended health behavior changes are offered as a means to scoping adjustments for DCM, and making broader recommendations for the men’s eHealth field. Our hypothesis—increased DCM exposure levels would be positively associated with men’s recent and intended health behavior changes—was supported by the study results. These findings corroborate broader acceptability claims and previous reports [7,14-16] about the compatibility of diverse eHealth resources with some men’s help-seeking preferences and practices. Also reinforced are assertions about the associations between engagement (based on self-reported duration and frequency of use) and men’s recent and intended health behavior changes [22]. That these findings held when controlling for key demographics synonymous with social determinants of health (ie, income, employment, education) might be interpreted as reflecting the wide reach, accessibility, and engagement potential of DCM for men from diverse backgrounds. In essence, the DCM content and dissemination strategies seem to be acceptable to, and engaging of men from an array of circumstances, with the net result that the DCM components can support health behavior changes in wide-ranging end users. Future work might use structural equation modeling to investigate statistically significant predictor variables including age, education, employment, and household income to distil their mediation and moderating effects. That said, it is also important to acknowledge the possibility, within the context of this study, that motivation to make health behavior changes actually caused some men to engage with DCM.

In further breaking down this study’s findings, questions emerge about if (and if so—how?) to act on some recent and intended health behavior change results to adjust the DCM content. Central here are the determinations for integrating specialist resources to DCM as a means to engaging more men with specific health behavior changes. For example, tobacco reduction and smoking cessation (TRSC) demand specialist resources (beyond the rhetoric messaging that *smoking is bad for you*) [15], and this study’s findings regarding low recent and intended changes for *Quit or reduce smoking* likely reflect the relatively small number of male smokers in Canada (compared with the overall population), as well as the lack of dedicated DCM resources focused on men’s TRSC. In essence, TRSC messaging was relevant to fewer end users (ie, smokers) and that relatively small subset of respondents were unlikely to have accessed DCM with the sole focus of reducing or quitting smoking. Similarly, that *Reduce stress* and *Improve consistent sleep quality* featured prominently as the most intended health behavior changes with fewer end users reporting recent changes in those health behaviors might indicate the need to adjust and/or integrate additional tailored DCM stress reduction and sleep aiding resources. Building on this point, Yardley et al [37] has argued the value of promoting effective engagement (defined empirically as sufficient engagement with the intervention to achieve intended outcomes) rather than simply more engagement. This is salient advice both in planning to adjust, add, and/or replace some DCM content and evaluating end users’ experiences through triangulating time-based exposure data with qualitative interviews as a means to more fully contextualizing men’s engagement. In addition, it is clear that longitudinal research is required to map content and end-user behavior changes over time and empirically guide ongoing adjustments to men’s eHealth programs.

Although acknowledging the inherent complexities to accounting for human behaviors and health behavior change in men more specifically [38], this study’s findings, consistent with findings from previous work [27,31], were mapped to the transtheoretical model (or stages of change). DCM respondents (contrasting the benchmark cohort) were clearly nestled across the contemplative, preparation, and action stages. That almost 10% and 13% of the variation in respondents' recent and intended health behaviour changes, respectively, were explained by DCM exposure levels and demographic variables, confirms the acceptability of, and engagement with DCM as well as the end users’ readiness to change. Herein, DCM users can be broadly characterized as planning to make, as well as investing actions toward some health behavior changes. This finding confirms the DCM end users as a distinct subset of the male Canadian population, and although challenges remain for advancing more men past the precontemplative stage (toward DCM or similar), the DCM end users offer unique opportunities for building engagement, and by extension, aiding some men’s efforts to maintain their health behavior changes.

### Limitations

A methodological limitation suggesting caution for interpreting the results of this study is the high potential for familywise errors as a byproduct of conducting 22 separate regression analyses. Self-report biases, both in relation to respondents recalling their DCM usage and disclosing recent and intended health behavior changes are also limitations. In particular, social desirability especially pertaining to exercise and healthy eating may have influenced men’s responses [39]. Reliance on quantitative measures limits the understanding about the diverse contexts that can influence men’s health practices including their eHealth help-seeking. In addition, that respondents were Canadian reduces the generalizability of the findings to other men living elsewhere. Further acknowledged is that the benchmark sample was not stratified by race or ethnicity because defining stratification quotas by race/ethnicity in addition to gender, age, and location would have created too many interlocking stratification variables to administer and lead to sparse data within certain strata. This study, although purposefully differentiating acceptability and engagement, was also limited by its elementary conceptualization and formal evaluation of the acceptability of DCM [40]. Some of these limitations can, however, be addressed in future work by triangulating data collection to qualitatively build understandings about what constitutes and counts as engagement from end-user perspectives and more fully evaluating the multifaceted concept of acceptability [40], comparing men from other countries who visit DCM, and/or the use of larger sample sizes with noninterlocking strata for race/ethnicity. Although the hypothesis that increased DCM exposure levels were positively associated with men’s recent and intended health behavior changes was supported by this study’s findings, RCTs and control group comparisons are needed to make dose-response and attributions claims to advance the men’s eHealth field. The inclusion of an economic analysis and specific behavior change techniques to consider cost would also strengthen future DCM studies [41].

### Implications and Conclusions

Men’s eHealth programs operate across a continuum of working to replace, augment, and connect men to professional *in person* health care services. This study, although focused on DCM in reporting men’s recent and intended health behavior changes, confirms the potential of eHealth programs for aiding health behavior changes. Also offered are important empirical insights and approaches to designing content and evaluating men’s eHealth resources, and mapping end-user outcomes with the transtheoretical model. Taken together, this study design and findings offer some methodological guidance and empirical weight to advance the men’s eHealth field.

## Appendix

Multimedia Appendix 1 Full table - Logistic regressions between demographics and recent health changes.

Multimedia Appendix 2 Full table - Logistic regressions between demographics and intended health changes.

## Abbreviations

DCM: Don’t Change Much
eHealth: electronic health
TRSC: tobacco reduction and smoking cessation
VIF: variance inflation factor
