# Supplementary material for: Mapping Canadian Men’s Recent and Intended Health Behavior Changes Through the Don’t Change Much Electronic Health Program
Source: J Med Internet Res. 2020 May 15;22(5):e16174. doi: 10.2196/16174 (PMC7260660; doi:10.2196/16174)
Supplement: Multimedia Appendix 1 [file jmir_v22i5e16174_app1.docx]

Multimedia Appendix 1. Full table - Logistic regressions between demographics and recent health changes.

| ***Dependent***  ***variables***  *(Recent Health Change)* | ***Predictor Variables,* OR (95% CI)** | | | | | | | | | | | |
| --- | --- | --- | --- | --- | --- | --- | --- | --- | --- | --- | --- | --- |
|  | *Don’t Change Much level of exposure*  *(Ref = No exposure)* | | | *Age (Years)* | *Employment* | *Household composition* | | *Education* | *Visible minority* | *Sexual orientation* | *Household income*  *(Ref = $60,000 to $119,999)* | |
|  | *Limited*  *exposure* | *Low*  *exposure* | *High*  *exposure* |  |  | *Lives with partner* | *Lives with children* |  |  |  | *$59,999*  *or less* | *$120,000*  *or more* |
| Changed diet or  improved eating habits | 2.008 (1.541, 2.616)*** | 3.228 (2.588, 4.027)*** | 5.628 (3.932, 8.055)*** | 1.009 (1.002, 1.015)** | 1.136 (0.933, 1.382) | 1.091 (0.898, 1.326) | 0.967 (0.794, 1.177) | 0.792 (0.672, 0.933)** | 1.17 (0.91, 1.505) | 1.042 (0.807, 1.346) | 0.887 (0.734, 1.074) | 0.955 (0.776, 1.175) |
| Made an effort to sit  less and walk more | 1.892 (1.442, 2.481)*** | 3.079 (2.472, 3.835)*** | 3.39 (2.457, 4.678)*** | 1.018 (1.012, 1.025)*** | 1.109 (0.902, 1.363) | 0.952 (0.777, 1.166) | 1.073 (0.876, 1.313) | 1.058 (0.893, 1.253) | 1.07 (0.823, 1.391) | 1.077 (0.828, 1.402) | 1.063 (0.87, 1.299) | 1.358 (1.101, 1.676)** |
| Increased exercise, sports or physical activity | 1.822 (1.398, 2.375)*** | 2.064 (1.661, 2.564)*** | 3.439 (2.444, 4.839)*** | 0.992 (0.986, 0.999)* | 0.898 (0.743, 1.086) | 1.046 (0.865, 1.264) | 0.852 (0.703, 1.032) | 1.079 (0.921, 1.265) | 1.272 (0.997, 1.623) | 0.933 (0.727, 1.196) | 0.846 (0.703, 1.018) | 1.273 (1.042, 1.557)* |
| I haven’t made  any changes | 0.39 (0.256, 0.595)*** | 0.23 (0.154, 0.344)*** | 0.14 (0.065, 0.301)*** | 0.994 (0.986, 1.002) | 1.142 (0.893, 1.461) | 0.846 (0.662, 1.081) | 1.202 (0.934, 1.548) | 1.153 (0.936, 1.421) | 0.854 (0.618, 1.18) | 0.936 (0.671, 1.307) | 1.09 (0.857, 1.385) | 1.091 (0.834, 1.426) |
| Drank less alcohol | 1.486 (1.108, 1.994)** | 1.822 (1.439, 2.306)*** | 3.287 (2.375, 4.549)*** | 0.993 (0.986, 1) | 0.95 (0.765, 1.179) | 0.964 (0.778, 1.196) | 0.917 (0.735, 1.145) | 0.803 (0.668, 0.964)* | 0.935 (0.704, 1.241) | 1.294 (0.988, 1.693) | 1.031 (0.836, 1.272) | 0.876 (0.692, 1.109) |
| Had a routine check-up  or visit to doctor | 1.855 (1.399, 2.46)*** | 2.129 (1.693, 2.677)*** | 2.082 (1.497, 2.896)*** | 1.049 (1.041, 1.056)*** | 0.572 (0.466, 0.703)*** | 1.012 (0.823, 1.245) | 1.126 (0.916, 1.382) | 0.924 (0.777, 1.1) | 0.911 (0.69, 1.202) | 1.236 (0.943, 1.619) | 0.86 (0.7, 1.056) | 1.138 (0.919, 1.41) |
| Improved consistent  sleep quality | 1.383 (1.014, 1.885)* | 1.781 (1.397, 2.27)*** | 2.376 (1.696, 3.329)*** | 1.001 (0.993, 1.008) | 1.014 (0.807, 1.275) | 0.926 (0.738, 1.161) | 1.05 (0.835, 1.319) | 0.994 (0.822, 1.202) | 1.095 (0.822, 1.459) | 1.204 (0.906, 1.599) | 1.113 (0.892, 1.39) | 1.051 (0.826, 1.337) |
| Lost weight | 1.255 (0.949, 1.66) | 1.602 (1.284, 2.001)*** | 2.186 (1.59, 3.005)*** | 1.002 (0.995, 1.008) | 1.106 (0.902, 1.356) | 1.121 (0.916, 1.371) | 0.907 (0.74, 1.11) | 0.84 (0.709, 0.995)* | 0.936 (0.718, 1.219) | 1.091 (0.84, 1.416) | 0.928 (0.761, 1.131) | 1.095 (0.887, 1.352) |
| Reduced stress level | 1.035 (0.758, 1.415) | 1.651 (1.305, 2.089)*** | 1.945 (1.393, 2.716)*** | 1.003 (0.996, 1.01) | 0.884 (0.712, 1.097) | 0.934 (0.752, 1.16) | 1.245 (1.001, 1.549)* | 0.981 (0.818, 1.177) | 0.993 (0.749, 1.316) | 1.155 (0.875, 1.523) | 1.244 (1.007, 1.537)* | 0.981 (0.777, 1.24) |
| Quit or reduced smoking | 1.652 (1.162, 2.347)** | 0.944 (0.66, 1.35) | 0.82 (0.461, 1.458) | 0.988 (0.979, 0.997)** | 1.369 (1.025, 1.827)* | 1.068 (0.804, 1.418) | 0.808 (0.592, 1.104) | 0.447 (0.341, 0.585)*** | 0.811 (0.541, 1.215) | 1.249 (0.881, 1.77) | 1.316 (1.006, 1.721)* | 0.6 (0.413, 0.873)** |

Note: *p<.05; **p<.01; ***p<.001; separate multiple logistic regressions were conducted for each outcome variable with all predictor variables entered on the same step.
