# Supplementary material for: Mapping Canadian Men’s Recent and Intended Health Behavior Changes Through the Don’t Change Much Electronic Health Program
Source: J Med Internet Res. 2020 May 15;22(5):e16174. doi: 10.2196/16174 (PMC7260660; doi:10.2196/16174)
Supplement: Multimedia Appendix 2 [file jmir_v22i5e16174_app2.docx]

Multimedia Appendix 2. Full table - Logistic regressions between demographics and intended health changes.

| ***Dependent***  ***variables***  *(Intended Health Change)* | ***Predictor Variables,* OR (95% CI)** | | | | | | | | | | | | | | | | | | | | | |
| --- | --- | --- | --- | --- | --- | --- | --- | --- | --- | --- | --- | --- | --- | --- | --- | --- | --- | --- | --- | --- | --- | --- |
|  | *Don’t Change Much usage*  *(Ref = No exposure)* | | | | | *Age (Years)* | *Employment* | | *Household composition* | | | | *Education* | | *Visible minority* | | *Sexual orientation* | | *Household income*  *(Ref = $60,000 to $119,999)* | | | |
|  | *Limited*  *exposure* | *Low*  *exposure* | | *High*  *exposure* | |  |  |  | *Lives with partner* | | *Lives with children* | |  |  |  |  |  |  | *$59,999*  *or less* | | *$120,000*  *or more* | |
| Improve consistent  sleep quality | 3.018 (2.3, 3.959)*** | | 3.633 (2.905, 4.543)*** | | 4.019 (2.911, 5.547)*** | 0.992 (0.985, 0.999)* | | 1.003 (0.81, 1.242) | | 0.856 (0.693, 1.057) | | 0.973 (0.787, 1.204) | | 0.84 (0.703, 1.004) | | 1.311 (1.008, 1.706)* | | 1.081 (0.826, 1.416) | | 0.961 (0.781, 1.182) | | 1.019 (0.814, 1.275) |
| Change diet or  improve eating habits | 3.671 (2.805, 4.805)*** | | 2.612 (2.097, 3.254)*** | | 3.244 (2.356, 4.465)*** | 0.992 (0.985, 0.998)* | | 0.94 (0.765, 1.154) | | 0.973 (0.793, 1.194) | | 1.165 (0.951, 1.427) | | 0.826 (0.696, 0.98)* | | 1.261 (0.976, 1.629) | | 1.093 (0.84, 1.422) | | 0.866 (0.709, 1.058) | | 0.945 (0.762, 1.172) |
| Increase exercise, sports or physical activity | 2.707 (2.048, 3.579)*** | | 2.934 (2.336, 3.687)*** | | 3.649 (2.565, 5.191)*** | 0.994 (0.988, 1)* | | 1.029 (0.85, 1.245) | | 1.05 (0.868, 1.27) | | 1.048 (0.863, 1.273) | | 0.928 (0.79, 1.09) | | 1.295 (1.01, 1.661)* | | 1.001 (0.778, 1.287) | | 1.037 (0.86, 1.249) | | 1.109 (0.904, 1.361) |
| Make an effort to sit  less and walk more | 2.837 (2.168, 3.713)*** | | 3.253 (2.607, 4.061)*** | | 2.954 (2.144, 4.069)*** | 1.013 (1.006, 1.019)*** | | 0.748 (0.61, 0.918)** | | 0.921 (0.75, 1.13) | | 1.201 (0.979, 1.474) | | 0.816 (0.687, 0.969)* | | 0.858 (0.652, 1.128) | | 1.057 (0.809, 1.38) | | 0.973 (0.796, 1.189) | | 1.105 (0.891, 1.371) |
| I don’t intend to make  any changes | 0.223 (0.135, 0.369)*** | | 0.143 (0.089, 0.23)*** | | 0.162 (0.082, 0.321)*** | 1.005 (0.997, 1.013) | | 0.943 (0.743, 1.195) | | 0.801 (0.629, 1.02) | | 1.092 (0.847, 1.407) | | 1.132 (0.921, 1.392) | | 0.889 (0.642, 1.231) | | 0.767 (0.542, 1.085) | | 1.063 (0.839, 1.347) | | 1.177 (0.904, 1.534) |
| Reduce stress level | 2.401 (1.803, 3.197)*** | | 2.337 (1.844, 2.96)*** | | 4.282 (3.086, 5.941)*** | 0.983 (0.976, 0.99)*** | | 1.176 (0.934, 1.482) | | 0.882 (0.705, 1.104) | | 1.115 (0.892, 1.393) | | 0.906 (0.751, 1.093) | | 1.046 (0.79, 1.384) | | 1.007 (0.756, 1.342) | | 0.987 (0.792, 1.229) | | 0.936 (0.738, 1.189) |
| Lose weight | 2.641 (2.016, 3.458)*** | | 2.4 (1.932, 2.983)*** | | 1.976 (1.44, 2.713)*** | 1.005 (0.999, 1.011) | | 1.057 (0.872, 1.282) | | 1.201 (0.992, 1.455) | | 1.04 (0.857, 1.261) | | 0.8 (0.68, 0.939)** | | 0.867 (0.674, 1.114) | | 1.194 (0.929, 1.534) | | 0.904 (0.75, 1.091) | | 1.085 (0.886, 1.328) |
| Have a routine check-up or visit to doctor | 2.19 (1.624, 2.953)*** | | 2.024 (1.57, 2.61)*** | | 2.067 (1.443, 2.961)*** | 1.021 (1.013, 1.029)*** | | 0.899 (0.71, 1.139) | | 0.967 (0.764, 1.224) | | 1.018 (0.802, 1.294) | | 0.895 (0.734, 1.092) | | 1.238 (0.913, 1.678) | | 0.858 (0.623, 1.18) | | 1.059 (0.842, 1.332) | | 0.812 (0.627, 1.05) |
| Drink less alcohol | 1.677 (1.204, 2.336)** | | 1.901 (1.453, 2.489)*** | | 2.974 (2.077, 4.26)*** | 0.995 (0.987, 1.003) | | 1.01 (0.782, 1.304) | | 0.915 (0.71, 1.179) | | 0.858 (0.664, 1.108) | | 0.61 (0.491, 0.757)*** | | 1.228 (0.894, 1.687) | | 0.944 (0.679, 1.314) | | 0.731 (0.569, 0.941)* | | 1.058 (0.813, 1.376) |
| Quit or reduce smoking | 1.415 (0.932, 2.148) | | 0.829 (0.535, 1.286) | | 1.043 (0.559, 1.946) | 0.987 (0.977, 0.997)* | | 1.241 (0.896, 1.72) | | 0.706 (0.51, 0.978)* | | 0.9 (0.619, 1.306) | | 0.446 (0.324, 0.614)*** | | 0.664 (0.403, 1.095) | | 1.039 (0.688, 1.57) | | 1.236 (0.909, 1.68) | | 0.504 (0.312, 0.816)** |

Note: *p<.05; **p<.01; ***p<.001; separate multiple logistic regressions were conducted for each outcome variable with all predictor variables entered on the same step.
